# Workplace exposure to carbon dioxide during routine laparoscopy – is it safe?

**DOI:** 10.12688/f1000research.24230.2

**Published:** 2020-09-21

**Authors:** Malin af Petersens, Fritiof Andersson Fenger-Krog, Jan G. Jakobsson

**Affiliations:** 1 Uppsala University, Uppsala, Sweden; 2 Karolinska Institutet, Stockholm, Sweden; 3 Department of Anaesthesia & Intensive Care, Institution for Clinical Sciencies, Danderyds Hospital, Stockholm, 18288, Sweden

**Keywords:** Ambient air, Carbon dioxide, Laparoscopy, Minimally invasive surgery, Occupational exposure, Work place exposure

## Abstract

**Background: **Minimally invasive surgeries have increased dramatically during the last decades. Carbon dioxide (CO _2_) is the gas used for insufflation during laparoscopies, creating space and visibility. The CO _2 _leaks into ambient air through ports where instruments are inserted. If the CO _2 _reaches a certain concentration it affects personnel health. There are national occupational exposure limits (OEL) for CO _2_, including a level limit value (LLV) of 5000 ppm. We are not aware of any previous studies addressing occupational exposure to CO _2 _during laparoscopies. The aim of this study was to assess the compliance to national OELs for CO _2 _during laparoscopies.

**Methods: **A gas detector was placed in the breathing zone of personnel in the operating theatre. The detector measured CO _2 _concentrations every tenth minute during laparoscopies in three locations.

**Results: **During 27 laparoscopies, the measured CO _2 _reached a maximum concentration of 1100 ppm, less than one fourth of the LLV. Median CO _2_ concentration was 700 ppm.

**Conclusion:** Results show that the occupational exposure to CO _2 _during laparoscopies is well below set OELs. Our findings support personnel safety associated with routine use of CO _2 _during laparoscopies.

## Introduction

Minimally invasive surgical techniques aim to achieve surgical therapeutic goals with minimal trauma ^1^. Minimal invasive surgery (MIS) has increased dramatically and is today well-established for huge numbers of procedures. During all forms of MISs, e.g. classic laparoscopy, gas insufflation is the most commonly used technique to create enough space to allow surgery ^2^. The preferred, most commonly used, gas for insufflation is carbon dioxide (CO _2_) ^3^. Characteristics of a perfect gas for insufflation include being colorless, incombustible, easily soluble in blood, non-toxic, inexpensive and easily removed from the body. CO _2_ is the gas that best matches these characteristics. To establish a gaseous cushion, an insufflator is used to pump CO _2_ into the abdominal cavity or other surgical field. CO _2_ will leak into the ambient air from the cavity where the instruments later are inserted, hence the CO _2_ concentration in ambient air in the operating theatre may be elevated and thus potentially cause personnel health concern.

### Hypercapnia

Symptoms of acute hypercapnia include flushed skin, headaches and sweating. Higher CO _2_ concentrations in ambient air may also cause anxiety and dizziness. High levels may further cause confusion and shortness of breath and eventually dimmed sight, tremor, unconsciousness or even death ^4, 5^. The individual response to elevated CO _2_ concentrations in ambient air varies depending on the time of exposure and CO _2_ concentration ^4^.

Recent work suggests that chronic exposure to higher concentration of CO2 may cause negative health effects, potentially having effects on fertility ^6, 7^.

### Occupational exposure limits

To prevent ill health, many countries have provisions regarding the highest acceptable concentrations of air pollutants at workplaces. The highest acceptable average concentration of an air pollutant in workplace air, calculated as time weighted average is known as the occupational exposure limit (OEL). There are two often used OEL values, the level limit value (LLV) and the short-term exposure limit (STEL). LLV is the OEL value for exposure during a working day, normally eight hours. STEL is the OEL value for a reference period of 15 minutes exposure ^8^. The Swedish OELs are based on the EU’s binding OELs, which includes an LLV of 5000 ppm for CO _2_^9^. The US National Institute for Occupational Safety and Health (NIOSH) has a similar level ^10^. The LLV is binding, unlike the STEL for CO _2_ at 10000 ppm which is the recommended highest value ^8^.

Personnel workplace safety is of huge importance and OELs has been set to secure good working condition, securing personnel health. The workplace CO _2_ concentrations may constitute a safety risk. We are not aware of previous studies explicitly addressing the adherence to OELs in operating theatres (OT) during routine use of CO _2_ for insufflation during laparoscopies.

### Aim

The aim of this study was to assess the occupational exposure to CO _2_ in OTs during laparoscopies to verify the compliance to set national (Swedish) OELs.

## Methods

### Study design and context

This was an explorative, non-interventional study of CO _2_ concentrations in ambient air during laparoscopies conducted at Danderyd Hospital during October 2019. The CO _2_ concentration was measured at three locations: old general surgery ward (OGSW; n=2), new general surgery ward (NGSW; n=1) and day surgery unit (DSU; n=1). The ventilation differed between the locations. In the two older OTs, the air volume flow was 710 L/s (liter/second) and 650 L/s. The air volume flow in the new OT was 2160 L/s during surgeries and 100 L/s during basic ventilation. In the DSU, the air volume flow was between 720 and 2160 L/s.

### Surgeries

The laparoscopies included in this study were aggregated into three groups based on the type of surgery: cholecystectomies, hernia repairs and intestinal surgeries. Five groups (A-E) were created depending on the type of surgery and the location: cholecystectomy DSU (A), cholecystectomy NGSW (B), hernia repair NGSW (C), intestinal surgery OGSW (D), intestinal surgery NGSW (E) ( Table 1).

**Table 1.** Summary of characteristics and possible confounding factors of group A–E. Surgery duration is presented as mean and standard deviation. Intra-abdominal pressure, number of people in the operating theatre and carbon dioxide concentration are presented as median and range. Number of people in operating theater excludes the patient.

	Group	Location	Surgeries (n)	Observations (n)	IAP (mmHg)	People in OT (n)	Duration (min)	Gas detector CO _2_ (ppm)
Cholecystectomy	A	DSU	6	52	12 [12–12]	7 [5–7]	77 (19)	600 [400–600]
	B	NGSW	3	30	12 [12–12]	7 [6–7]	90 (26)	700 [600–1100]
Hernia repair	C	NGSW	4	40	12 [12–14]	6 [6–7]	90 (22)	700 [600–1000]
Intestinal surgery	D	OGSW	2	35	13 [12–14]	9 [7–10]	165 (120)	600 [600–1000]
	E	NGSW	5	57	14 [12–14]	7 [5–8]	113 (85)	700 [600–800]
**Total**			20	210				700 [400–1100]

*IAP* intra-abdominal pressure, *CO _2_* carbon dioxide, *OT* operating theatre, *OGSW* old general surgery ward, *NGSW* new general surgery ward, *DSU* day surgery unit, *ppm* parts per million

### Data collection

A gas detector ( **TM Dräger X-am 5600, Germany**) was used to record point measurements of CO _2_ concentrations during the surgeries. This detector has a measurement range of 0–5%, hence the full-scale value is 5% (50000 ppm). The manufacturer of the sensor states an accuracy of ±800 ppm if the CO _2_ concentration is 25000 ppm or less. The resolution of the sensor is 100 ppm, thus the scale is divided into 500 equal divisions (400 ppm, 500 ppm, 600 ppm etc.). The exact value displayed depends on the span value set during calibration.

The primary outcome of the study was the concentration of CO _2_ in ambient air during MIS. The gas detector was positioned at a height of 153 cm in the OT at the IV pole on the right side of the patient. The CO _2_ concentration was noted manually every tenth minute starting on the hour. Observations were collected from the point measurement before the start of surgery until the point measurement after the end of surgery. Start and end of surgery were determined by start- and endpoint as noted by the nurse anesthetist in the medical record.

One of the secondary outcomes was the CO _2_ concentration at different heights in the OT. During a laparoscopic hernia repair in the NGSW (group C) the gas detector was placed as previously described. During the first two observations (20 minutes) the detector was placed at a height of 153 cm and was then moved to 105 cm for the next two observations. The following two observations were collected at a height of 15 cm and the detector was then moved back to a height of 153 cm. Observations were collected by changing the height as described every 20 minutes until the end of surgery.

The other secondary outcome of the study was the maximum concentration of CO _2_ when gas is allowed to freely enter the OT by disconnecting the insufflation tube from the insufflator. This was conducted in an OT in the NGSW. The gas detector was placed as described previously. The insufflator was set at high flow and the intra-abdominal pressure (IAP) was set to 14 mmHg. The central gas was turned on for five minutes and the highest observed CO _2_ concentration, the CO _2_ concentrations at the beginning and end of the attempt were noted manually. The attempt was conducted three times, the first time with basic ventilation and the third time with operation ventilation.

### Statistical analysis

Data is presented as mean, standard deviation, or median and range as applicable. For descriptive analysis and Kruskal-Wallis test, Microsoft Excel (version 16.32, 2019) was used. Descriptive analysis was performed to show measured CO _2_ concentrations, characteristics and possible confounding factors among the five groups. Kruskal-Wallis test was used to analyze if there was a significant difference among the three different heights where the CO _2_ concentration was measured. A *P* value <0.05 was considered statistically significant. To illustrate CO _2_ concentrations in different groups, box plots were created using R programming language (version 3.6.1, 2019-07-05).

### Ethical considerations

This was an explorative non-interventional air quality study and the CO _2_ concentrations in OTs were monitored only to ensure personnel health. Personnel safety and health is of great importance and this study was significant to assess the personnel safety related to CO _2_ in OTs. No patient or personnel data were collected. There was no need for ethical approval. The Head of the Department of Anesthesia and Intensive Care as well as the Head of the Department of Surgery approved the study.

## Results

The CO _2_ concentration was measured during 20 surgeries where a total of 210 observations were collected with the gas detector. The number of observations in each group ranged from 30 to 57. Possible confounding factors such as the number of people in the OT and IAP showed little variation between groups ( Table 1).

**Figure 1.**
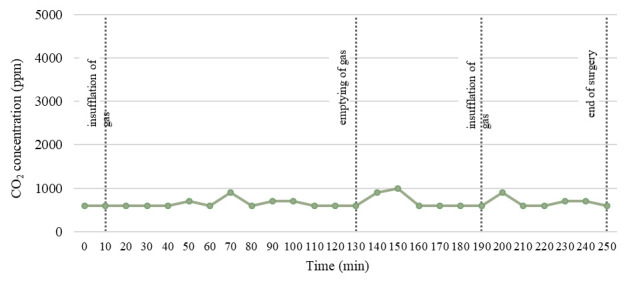
CO2 concentration measured during one entire laparoscopic procedure. (minutes on x-axis and CO2 concentration ppm on Y-axis.

During the surgeries, the measured CO _2_ concentration showed minor variation. Point measurements from one of the surgeries were selected to show an example of intraoperative variation of CO _2_ concentration measured with the gas detector ( Figure 1). During this surgery, 65% of the measured CO _2_ concentrations were at 600 ppm and observations ranged from 600 to 1000 ppm. Variation of intraoperative CO _2_ concentration was occasionally coherent with emptying and insufflations of gas in the peritoneal cavity. All measured CO _2_ concentrations during this surgery were less than or equal to 20% of the LLV ( Figure 1).

**Figure 2.**
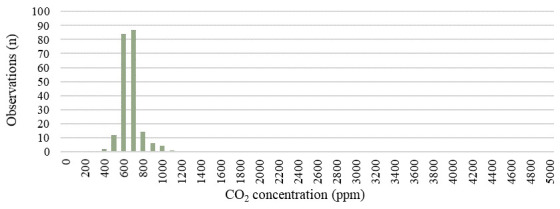
Measured CO2 concentrations during surgery. CO2 concentration measured on x-axis, number of observations on y-axis.

Out of all observations collected in groups A-E, none exceeded 1100 ppm ( Figure 2). The CO _2_ concentration was 600 ppm or 700 ppm in 81% of the observations ( Figure 2). Concentration of CO _2_ exceeding 700 ppm was seen in 12% of observations.

**Figure 3.**
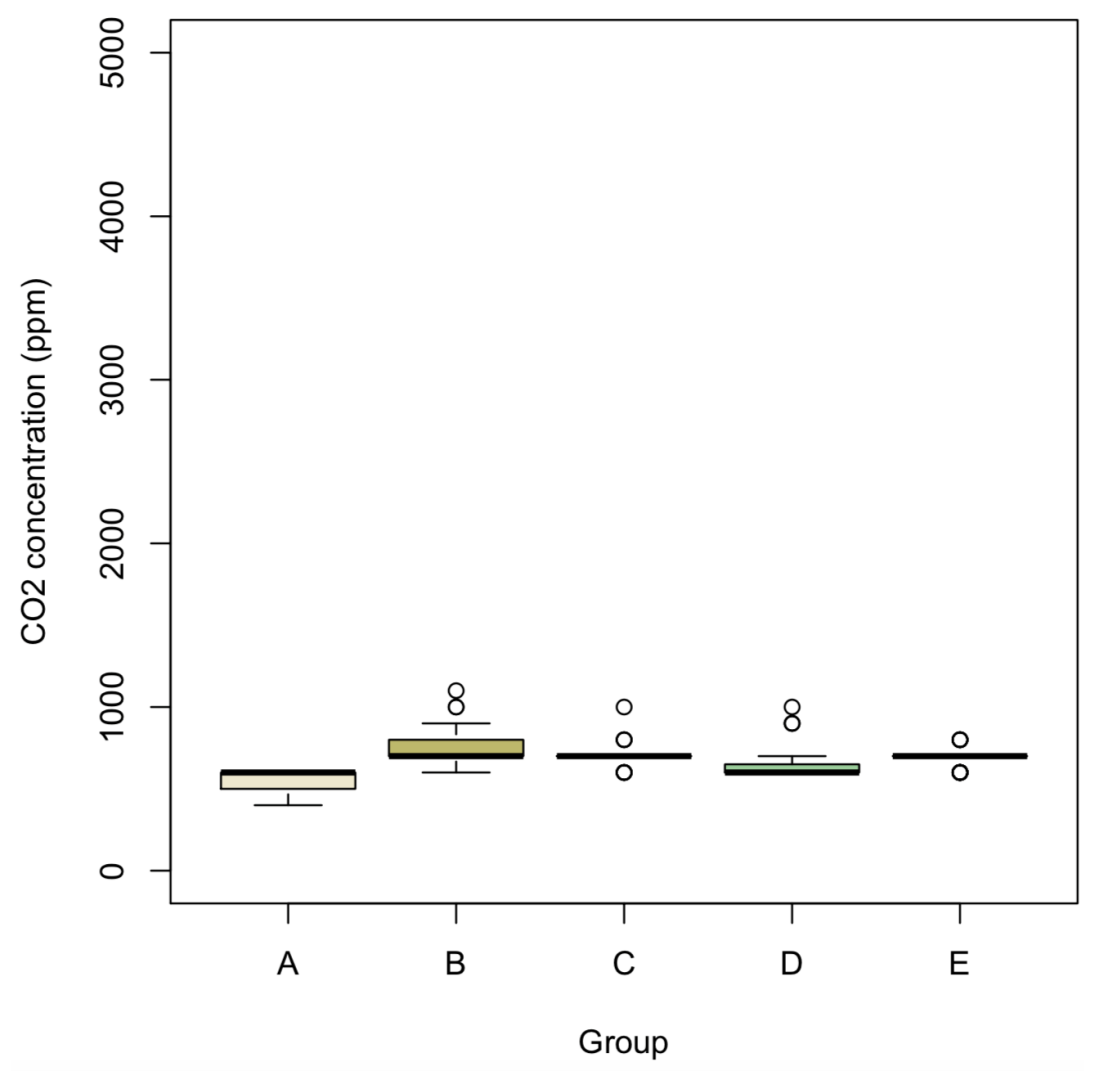
Box plots for CO2 concentration measured during the 5 different surgical procedures. Procedures on x-axis, CO2 concentration on y-axis. A and B cholecystectomy; A Days surgical unit, B in News Surgical unit, C Hernia repair in new surgical unit, D and E Intestinal surgery; D in old surgical unit, E in new surgical unit.

The CO _2_ concentration during the surgeries was measured at 400–1100 ppm and never exceeded 22% of the LLV at 5000 ppm ( Figure 3). Because of the scarce variation in all the groups, sporadic variations are frequently shown as outliers in the box plot.

When the CO _2_ concentration was measured at different heights in the OT, results of Kruskal-Wallis test showed no significant difference between heights (χ ^2^ = 1.371, p = 0.504).

**Figure 4.**
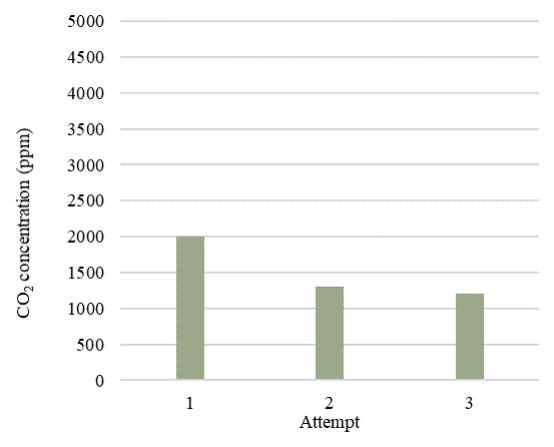
CO2 concentration peak value measured during CO2 release into the operating theater. Attempt on x-axis and peak CO2 concentration ppm on y-axis.

During the three attempts when CO _2_ was allowed to freely enter the OT for five minutes, the highest value measured was 2000 ppm. This value is a fifth of the STEL and less than half of the LLV ( Figure 4).

## Discussion

The primary aim of this study was to measure the concentration of CO _2_ in ambient air during laparoscopies to verify compliance to the set national OELs. During 20 laparoscopies in three different locations, the measured CO _2_ concentration did not exceed 1100 ppm, which is less than one fourth of the LLV and one ninth of the STEL. Furthermore, when gas was allowed to freely enter the OT for five minutes, mimicking an accidental user error, the measured CO _2_ reached a maximum concentration of two fifths of the LLV. Thus, all measured CO _2_ concentrations were well below set OELs, hence the findings are reassuring. Our measured vales must be put into perspective. Ambient air CO _2_ concentration is today higher than ever before, the global average atmospheric carbon dioxide in 2019 was 409.8 parts per million (ppm for short), with a range of uncertainty of plus or minus 0.1 ppm.

Personnel health is of great importance and it is the obligation of all healthcare organizations to secure proper workplace safety including ambient air quality. However, we are not aware of previous studies reporting CO _2_ concentrations during laparoscopies. Air quality indices including CO _2_ concentrations have nonetheless recently been studied during other types of MIS in a gastrointestinal endoscopy unit ^11^. Similar to our findings, the CO _2_ concentration in the procedural area was well below set OELs with a median concentration of 593.1 ppm (range 400–1645.9 ppm).

In our study we measured CO _2_ as a direct pollutant. Conversely, like the study in the gastrointestinal endoscopy unit, the CO _2_ concentration in other hospital environments has previously been studied as an indicator of air quality rather than as a direct pollutant ^12– 14^. The ventilation must thus be taken into account. The ambient air average concentration is today high. Additional CO _2_ load, the amount of CO _2_ added to the ambient air, CO _2_ exhaled by subjects in the room and CO _2_ from any additional sources, (e.g. from the insufflation of CO _2_ gas) and air change, ventilation, are the main factors for secure ambient air quality. CO _2_ as an indicator of air quality has also been studied in other environments such as classrooms ^15, 16^. Overall, results in these studies of hospital environments and classrooms show concentrations considerably lower than the set OELs. The hospital operating room ventilation is most effective as was shown in our testing extensive leakage, caused by a simulated user error.

The results must be put in perspective of some limitations. The gas detector can only assume certain fixed values, thus small changes in concentration were not detected. Nevertheless, it was important in this study to distinguish between measured CO _2_ concentrations and national OELs and detection of smaller changes in CO _2_ concentration were not needed for this purpose. The device was calibrated but the accuracy of the instrument must also be acknowledged. Still even in a worst case scenario for accuracy the measured levels are well below OEL.

Possible confounding factors include the number of trocars, the ventilation systems, conversion to open surgery, the number of people in the OT and the IAP. Nevertheless, the CO _2_ concentration varied little among groups and thus these factors did not seem to have a considerable impact on the concentrations in this study.

Data was handled manually due to the inability to store data and the inability to transfer data to a computer in the gas detector. Concentrations at different heights were only measured during one surgery and differences between heights can therefore not be established. Moreover, concentrations were only measured at one hospital and during rather few surgeries and results may not be generalizable to other hospitals, although all concentrations in the different locations were very low. CO _2_ concentrations were only studied at adult surgery departments and because of differences in abdominal cavity volume, amount of CO _2_ used and other unrecognized factors, results may be less transferable to children.

In addition, we looked solely at laparoscopies in this study. Other MIS techniques, such as colonoscopies and robotic surgeries, can be performed in other environments or have a longer duration which might affect CO _2_ concentrations.

## Conclusion

This study shows that the occupational exposure to CO _2_ in OTs during laparoscopies is well below set OELs. Our findings also suggest that CO _2_ concentrations are distributed the same way at different heights in the OT. Even when gas is freely entering the OT for five minutes, mimicking an accidental user error, the CO _2_ concentrations are well below OELs, hence the results are reassuring. Our findings support personnel safety associated with routine use of CO _2_ for insufflation during laparoscopy.

## Data availability

### Underlying data

Open Science Framework: CO2 measurement, https://doi.org/10.17605/OSF.IO/6S5UQ ^17^.

Data are available under the terms of the Creative Commons Zero "No rights reserved" data waiver (CC0 1.0 Public domain dedication).
