# Supplementary material for: Workplace exposure to carbon dioxide during routine laparoscopy – is it safe?
Source: F1000Res. 2020 Sep 21;9:571. Originally published 2020 Jun 8. [Version 2] doi: 10.12688/f1000research.24230.2 (PMC7520708; doi:10.12688/f1000research.24230.2)
Supplement: Peer review history

# Peer review: Reviewer response for version 2

**DOI:** 10.5256/f1000research.29591.r71740

**Published:** 2020-09-25

**Authors:** Jakob Walldén

**Affiliations:** 1 Anaesthesia and Intensive Care Section, Umeå University, Umeå, Sweden; 2 Anesthesia and Intensive Care, Sundsvall Hospital, Sundsvall, Sweden

The authors have adressed the issues pointed out in the revision.

Is the work clearly and accurately presented and does it cite the current literature?

Yes

If applicable, is the statistical analysis and its interpretation appropriate?

Yes

Are all the source data underlying the results available to ensure full reproducibility?

Yes

Is the study design appropriate and is the work technically sound?

Yes

Are the conclusions drawn adequately supported by the results?

Yes

Are sufficient details of methods and analysis provided to allow replication by others?

Yes

Reviewer Expertise:

Anesthesiology

I confirm that I have read this submission and believe that I have an appropriate level of expertise to confirm that it is of an acceptable scientific standard.

# Peer review: Reviewer response for version 2

**DOI:** 10.5256/f1000research.29591.r71739

**Published:** 2020-09-22

**Authors:** Colin F. Royse

**Affiliations:** 1 Department of Surgery, University of Melbourne, Parkville, Victoria, Australia

Amendments are satisfactory.

Is the work clearly and accurately presented and does it cite the current literature?

Yes

If applicable, is the statistical analysis and its interpretation appropriate?

Yes

Are all the source data underlying the results available to ensure full reproducibility?

No source data required

Is the study design appropriate and is the work technically sound?

Yes

Are the conclusions drawn adequately supported by the results?

Yes

Are sufficient details of methods and analysis provided to allow replication by others?

Yes

Reviewer Expertise:

Anesthesiology, Echocardiography, cardiac surgery outcomes, Quality of recovery after surgery

I confirm that I have read this submission and believe that I have an appropriate level of expertise to confirm that it is of an acceptable scientific standard.

# Peer review: Reviewer response for version 1

**DOI:** 10.5256/f1000research.26727.r70136

**Published:** 2020-09-11

**Authors:** Jakob Walldén

**Affiliations:** 1 Anaesthesia and Intensive Care Section, Umeå University, Umeå, Sweden; 2 Anesthesia and Intensive Care, Sundsvall Hospital, Sundsvall, Sweden

This is a study evaluating carbon dioxide levels during laparoscopies in operating theaters in relation to occupational exposure limits. The study design and results are clear and easy to understand and the conclusions are sound in relation to the results- carbon dioxide levels are not a health related issue.

 However there are a few minor issues I would like to address to improve the manuscript:

- The levels of carbon dioxide detected are low. What are normal background levels? Outdoor, indoor, operating rooms with high ventilation? Please relate your findings and include in the discussion.
- How is CO2 levels affected by the the high ventilation? Is it even possible to detect high levels, are CO2 washed out quickly? Consider including the issue in discussion.
- The accuracy of the method is stated as +/- 800 ppm. This means that there is a possibility that the levels detected at 800 ppm is in the range 0-1600 ppm. The rise in CO2 during flushing shows that sensor work. However, if related to the primary aim, even if the real CO2 levels are in the higher interval, it is far below OEL. Please include briefly in discussion.
- Please use L/min instead of l/min.

Is the work clearly and accurately presented and does it cite the current literature?

Yes

If applicable, is the statistical analysis and its interpretation appropriate?

Yes

Are all the source data underlying the results available to ensure full reproducibility?

Yes

Is the study design appropriate and is the work technically sound?

Yes

Are the conclusions drawn adequately supported by the results?

Yes

Are sufficient details of methods and analysis provided to allow replication by others?

Yes

Reviewer Expertise:

Anesthesiology

I confirm that I have read this submission and believe that I have an appropriate level of expertise to confirm that it is of an acceptable scientific standard, however I have significant reservations, as outlined above.

# Response

**Published:** 2020-09-11

**Authors:** Jan Jakobsson

**Affiliations:** Department of Physiology and Pharmacology, Karolinska Institutet, Sweden

Dear Referee.

 Thank you for important comments.

- The average ambient air CO2 has passed 400 ppm and is today higher than ever before.  
- Operating rooms are indeed well ventilated. Merely a rather limited increase was seen in the operating room CO2 concentration when a 5 minute free release of CO2 accident, user error, was mimicked  
- The instrument used was calibrated but the accuracy must of course be acknowledged. 

We have now revised and incorporated these important aspects into the discussion.

# Peer review: Reviewer response for version 1

**DOI:** 10.5256/f1000research.26727.r65631

**Published:** 2020-07-13

**Authors:** Colin F. Royse

**Affiliations:** 1 Department of Surgery, University of Melbourne, Parkville, Victoria, Australia

The authors have conducted a safety study to measure the Carbon Dioxide concentration that could be inhaled by staff in the operating room during laparoscopic surgery.

 They chose three different locations, each of which have different ventilation and air volume turnover methodologies. They conducted serial assessments over time for multiple surgeries. They also conducted a brief experiment where they ran CO _2_ freely into the operating room.

 They found that there is little variance between each of the operating locations or types of surgery. Importantly, all will well below the acceptable limits (level limit value of 5000 PPM). It was interesting, that even the range of CO _2_ was relatively small, with the maximum amount being around 1100 PPM. When CO _2_ is freely introduced into the air, the values were higher-approximating 2000 PPM, which was still well below the level limit value that is mandated in Europe.

 This paper adds to our literature of occupational safety. The use of different theatre locations (old and new) with different air volume turnover rates allows this knowledge to be transferred to other locations and hospitals. It is highly unlikely, even with old theatres, that laparoscopic surgery will produce high levels of carbon dioxide, sufficient to affect health care workers.

 The conclusion is consistent with the data, and supports that even an accidental user error, where carbon dioxide is freely running into the theatre, is still unlikely to cause a health issue for workers-this is very encouraging data.

Is the work clearly and accurately presented and does it cite the current literature?

Yes

If applicable, is the statistical analysis and its interpretation appropriate?

Yes

Are all the source data underlying the results available to ensure full reproducibility?

No source data required

Is the study design appropriate and is the work technically sound?

Yes

Are the conclusions drawn adequately supported by the results?

Yes

Are sufficient details of methods and analysis provided to allow replication by others?

Yes

Reviewer Expertise:

Anesthesiology, Echocardiography, cardiac surgery outcomes, Quality of recovery after surgery

I confirm that I have read this submission and believe that I have an appropriate level of expertise to confirm that it is of an acceptable scientific standard.
